# Hydrogen Sulfide in Physiology and Pathogenesis of Bacteria and Viruses

**DOI:** 10.1002/iub.1740

**Published:** 2018-03-30

**Authors:** Virender Kumar Pal, Parijat Bandyopadhyay, Amit Singh

**Affiliations:** Department of Microbiology and Cell Biology, Centre for Infectious Disease Research, Indian Institute of Science (IISc), Bangalore, India

**Keywords:** hydrogen sulfide, cytoprotectant, antioxidant, metabolism, infection, Mycobacterium tuberculosis, HIV

## Abstract

An increasing number of studies have established hydrogen sulfide (H_2_S) gas as a major cytoprotectant and redox modulator. Following its discovery, H_2_S has been found to have pleiotropic effects on physiology and human health. H_2_S acts as a gasotransmitter and exerts its influence on gastrointestinal, neuronal, cardiovascular, respiratory, renal, and hepatic systems. Recent discoveries have clearly indicated the importance of H_2_S in regulating vasorelaxation, angiogenesis, apoptosis, ageing, and metabolism. Contrary to studies in higher organisms, the role of H_2_S in the pathophysiology of infectious agents such as bacteria and viruses has been less studied. Bacterial and viral infections are often accompanied by changes in the redox physiology of both the host and the pathogen. Emerging studies indicate that bacterial-derived H_2_S constitutes a defense system against antibiotics and oxidative stress. The H_2_S signaling pathway also seems to interfere with redox-based events affected on infection with viruses. This review aims to summarize recent advances on the emerging role of H_2_S gas in the bacterial physiology and viral infections. Such studies have opened up new research avenues exploiting H_2_S as a potential therapeutic intervention.

## Introduction

Early life forms first appeared on an anoxic earth in the Archean eon, approximately 3.8 billion years ago (1,2). Among them, were the dissimilatory sulfate-reducing bacteria which constitute one of the oldest forms of bacterial life on earth. These bacteria utilized inorganic sulfur substrates and produced hydrogen sulfide (H_2_S) as the end product of anaerobic respiration (3). Before the “great oxidation event” which occurred 2.5 billion years ago leading to an increase in atmospheric oxygen, H_2_S remained the most abundant and versatile chemical on the primitive earth (4,5). In fact, H_2_S is widely believed to be the primordial sustainable energy source (6). Primitive photolithotrophs used sulfide as the terminal electron acceptor, similar to today’s green and purple sulfur bacteria. Therefore, sulfide-based metabolism may have preceded the present, oxygen-based life on the planet by billions of years (7–9).

**Fig 1:**
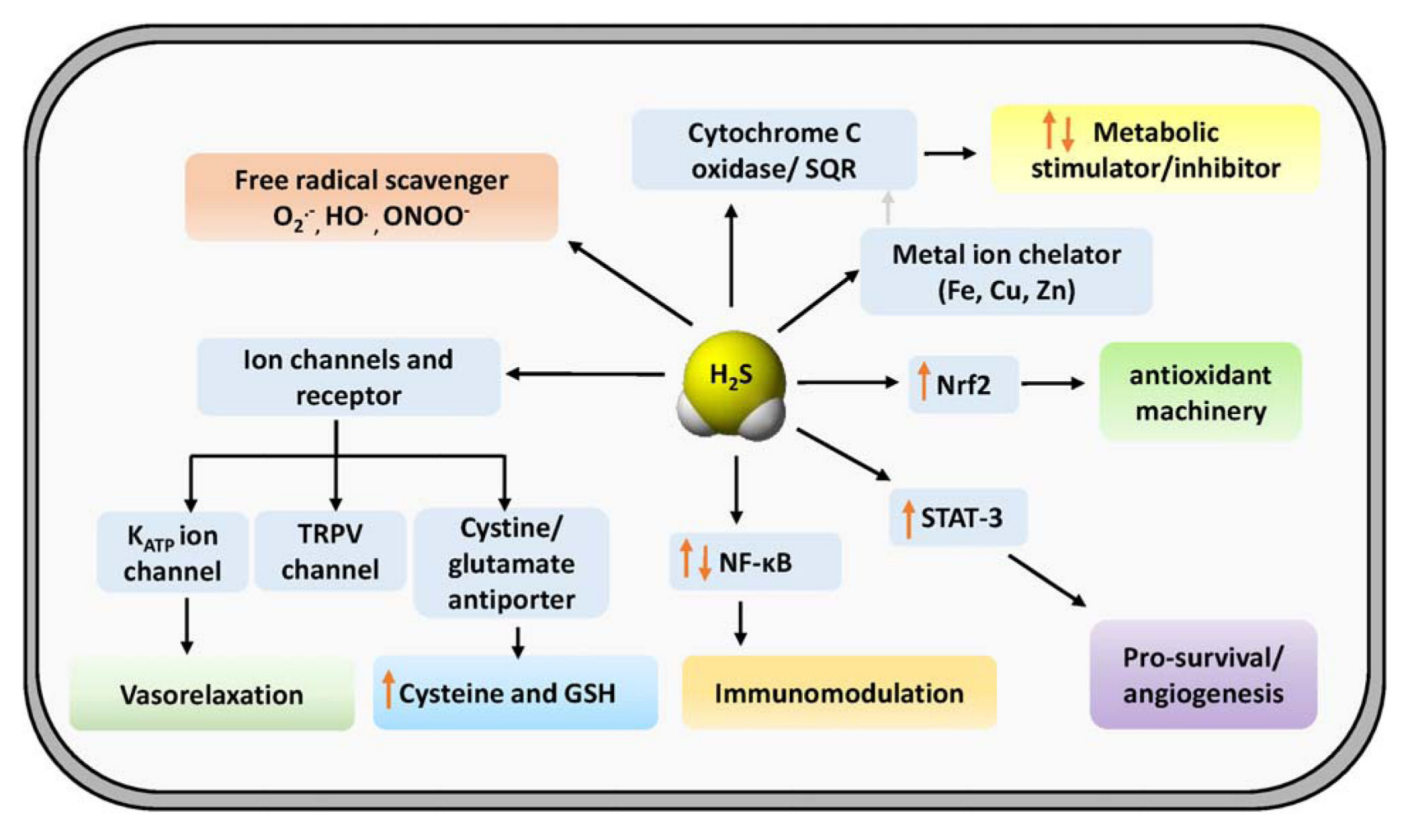
Molecular targets of H_2_S in mammalian system. Top left: H_2_S has the ability to directly scavenge reactive oxygen and nitrogen species (ROS/RNS). Top right: H_2_S targets metal cofactor of cytochrome c oxidase and leads to inhibition of cellular respiration. Oxidation of H_2_S by sulfide quinone oxidoreductase (SQR) couples catabolism of H_2_S with mitochondrial electron transport chain (ETC) and thus modulates cellular metabolism. Bottom left: ion channels which are involved in systemic responses to H_2_S in blood vessels, heart, and neurons. Bottom right: H_2_S targets cysteine thiols by S-persulfidation of intracellular signaling proteins and transcription factors which likely accounts for the downstream effects on inflammation, antioxidant response, cellular proliferation, and survival.

In sharp contrast to its pivotal role early in the evolutionary timeline, H_2_S is known mostly for being a foul smelling poisonous gas, associated with sewers, septic tanks, and as a weapon of chemical warfare during the First World War. Consequently, majority of the research pertaining to this gas has been conducted from a toxicology point of view (10,11). With studies published as long back as 1803 highlighting the detrimental effect of H_2_S on animals, along with the recent gene expression data, H_2_S was condemned as a respiratory and metabolic poison (12). It was not until the 1940s that the “transsulfuration” pathway involving the production of H_2_S by interconversion between cysteine, homocysteine via cystathionine was described for the first time in liver homogenates (13,14). Further studies led to detailed biochemical characterization of the enzymes cystathionine beta synthase (CBS) and cystathionine gamma lyase (CSE) involved in the transsulfuration reaction. Later, another enzyme, 3-mercaptopyruvate sulfurtansferase (3-MST) was identified as a part of H_2_S biogenesis pathways (15–18). However, the functional implication of the H_2_S biogenesis remained elusive for a long time. First glimpse of H_2_S involvement in cellular physiology emerged from the studies demonstrating measurable levels of endogenous H_2_S within brain tissues of healthy individuals (0.65–0.73 mg/g) and animals (1.57 ± 0.04 μg/g) (19–21). Along these lines, higher levels of neuronal H_2_S was found to be due to greater expression of CBS in the brain tissues. Additionally, H_2_S production in the brain tissue was efficiently reduced using pharmacological CBS inhibitors (hydroxylamine and amino-oxyacetic acid). Further studies proposed that H_2_S facilitates the induction of hippocampal long-term potentiation (LTP) by enhancing the activity of *N*-methyl d-aspartate (NMDA) receptors (22). Later, H_2_S was found to relax vascular smooth muscle by activating ATP-sensitive K^+^, intermediate conductance Ca^2+^ sensitive K^+^, and small conductance Ca^2+^-sensitive K^+^ channels (23–25). Importantly, H_2_S was identified to protect from oxidative stress and ischemia-reperfusion injury by multiple mechanisms such as restoring the levels of GSH and direct scavenging of mitochondrial ROS (Fig. 1) (26,27). These discoveries further led to the disclosure of mechanisms by which H_2_S protects various organs, including the heart and kidney from oxidative stress and ischemia-reperfusion injury (28). Based on these studies, H_2_S was inducted as the newest member of the family of small molecule gaseous transmitters or “gasotransmitters” alongside nitric oxide (NO) and carbon monoxide (CO) (29). Along this line hydrogen gas (H_2_) has also emerged as a potential gaseous signaling molecule with therapeutic antioxidant function (30,31). Recently, H_2_S was found to have a protective role in airway epithelial cells infected with respiratory syncytial virus (RSV) demonstrating for the first time that this molecule might be used as a therapeutic agent. Needless to say, the role of H_2_S has permeated to areas of metabolism, redox physiology, neurophysiology, apoptosis, angiogenesis, ageing, inflammation, atherosclerosis, pulmonary diseases among others with a whole spectrum of physiological implications (32–34).

H_2_S producing bacteria were discovered way back in 1877. Many investigators demonstrated bacterial production of H_2_S by its rotten egg smell and its ability to react with lead acetate resulting in the blackening of paper strips impregnated with lead acetate (12,35). In fact, lead acetate test was successfully exploited to distinguish between the paratyphoid and enteritidis groups and still remains an indispensable diagnostic tool (36). In the area of marine microbiology, H_2_S emitted from deep sea vents is often referred to as the “sunlight of the deep ocean” (37,38). H_2_S forms an important source of metabolic energy for the microorganisms inhabiting such niches, reminiscent of the primordial earth (6). Many bacterial species were demonstrated to possess orthologs of the transsulfuration pathway enzymes (CBS and CSE) and of 3-MST (36,39–41). However, the significance of H_2_S biogenesis in bacteria remained poorly characterized. In 1960s, co-culture experiments with *Desulfovibrio desulfuricans*/*Pseudomonas aeruginosa* and *Escherichia coli*/*Staphylococcus aureus* provided the first evidence of a possible “protective” role of H_2_S in bacteria. H_2_S produced by *D. desulfuricans* was demonstrated to be the “diffusible” factor responsible for imparting pseudomonads the ability to resist heavy metal (e.g., mercury) toxicity (42). Similarly, H_2_S produced by *E. coli* protected *S. aureus* from merbromin and mercuric chloride (43). Surprisingly, it took more than four decades to discover additional roles of H_2_S in protecting diverse bacterial species from oxidative stress and antibiotics (44). Similar reports of protective influence of H_2_S have emerged in plants and nematodes, however, they are beyond the scope of this review. Altogether, it appears that H_2_S is an important biological effector molecule with diverse roles in organisms ranging from bacteria to mammals.

**Fig 2:**
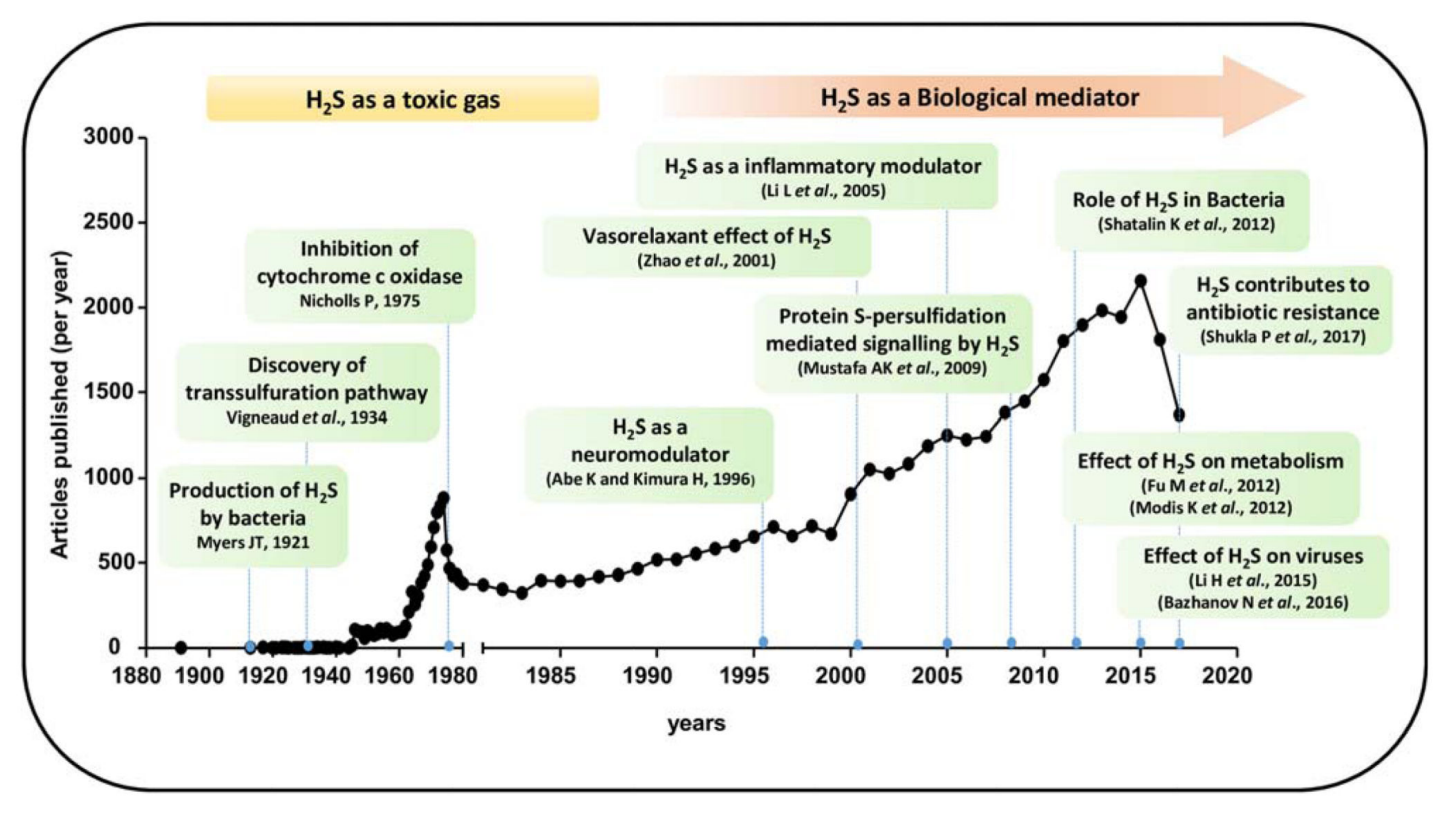
Survey of literature on H_2_S. The figure shows number of published articles per year till 2017 (source: PubMed). Some of the landmarks discoveries are highlighted in the figure inset.

While there has been a steady rise in the number of studies pertaining to the physiological role of H_2_S in mammalian systems, there is a significant lack in our understanding of the same when it comes to infectious agents like bacteria and viruses. A survey of PubMed also shows that the studies on H_2_S and bacterial/viral infections are relatively fewer as compared to mammalian systems (Fig. 2). Keeping this in mind, our effort is to compile a summary of the existing studies providing insights on how H_2_S exerts its biological influence on infectious diseases caused by bacterial and viral agents. First, we will provide a brief description of H_2_S biogenesis pathways and chemical properties of H_2_S. This will be followed by a description of studies on the contribution of H_2_S in influencing the physiology of bacteria and virus infected cells.

## Biogenesis of H_2_S

**Fig 3:**
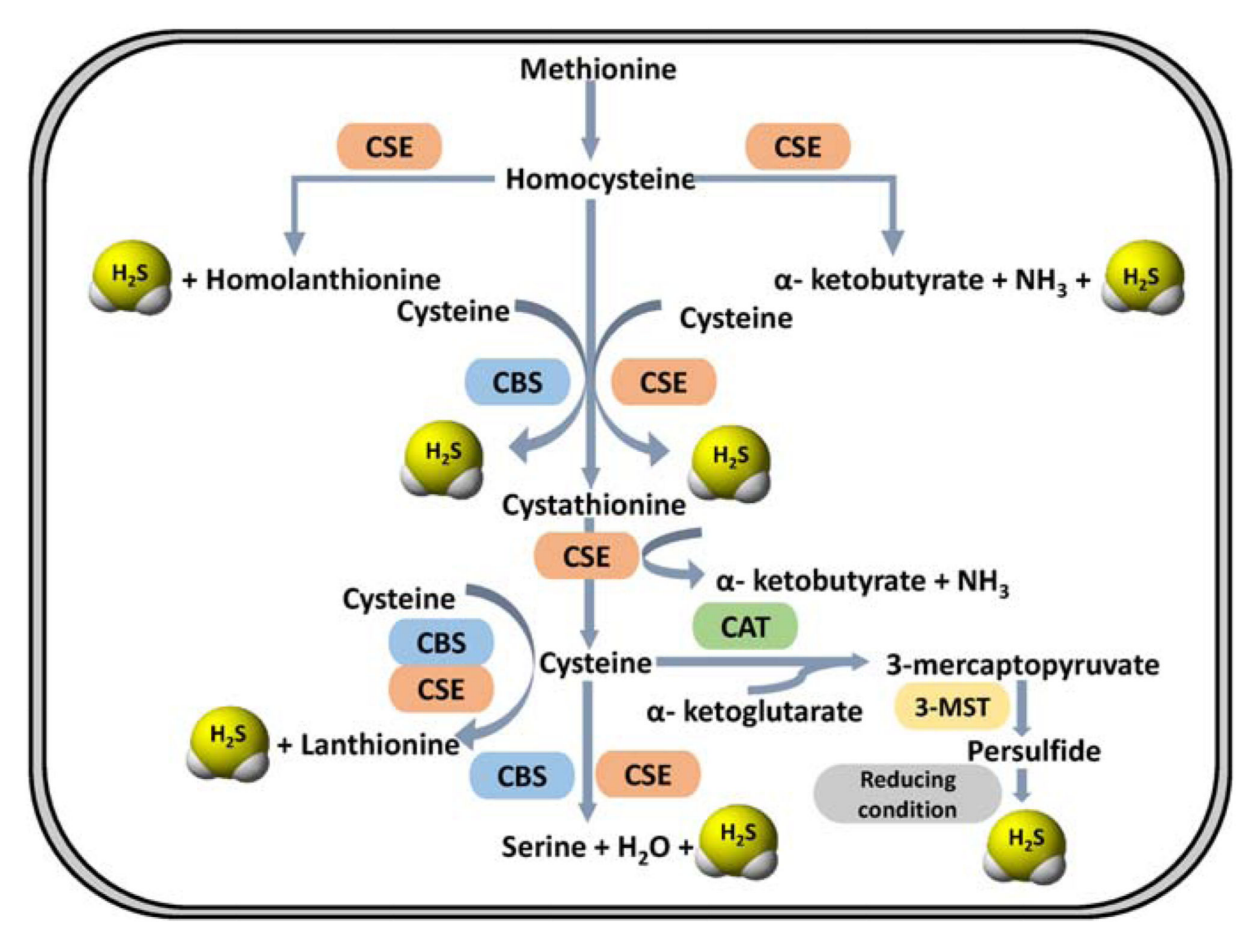
Pathways involved in enzymatic biogenesis of H_2_S. The transsulfuration pathway consisting of the enzymes cystathionine *β*-synthase (CBS) and Cystathionine *γ*-lyase (CSE) is the major pathway for biological H_2_S production. In addition to this 3-mercaptopyruvate sulfurtransferase/cysteine aminotransferase (3-MST/CAT) pathway also contribute significantly to the production of H_2_S.

The biogenesis of H_2_S has been mainly attributed to the transsulfuration pathway (45). This pathway has been known for many years and is evolutionarily conserved and present in many lower species as well as in mammals (46). Two enzymes constitute this pathway namely cystathionine beta synthase or CBS (EC 4.2.1.22) and cystathionine gamma lyase or CSE (EC 4.4.1.1). Both the enzymes uses pyridoxal l- phosphate (PLP) as cofactor and are hence sensitive to common PLP dependent enzyme inhibitors like hydroxylamine (47,48). Apart from transsulfuration, an additional pathway also exists which leads to the biogenesis of H_2_S. Cysteine aminotransferase (CAT, EC 2.6.1.3) catalyses the reaction of cysteine with keto acids (e.g., *α*-ketoglutarate) to form 3-mercaptopyruvate, which is subsequently desulfurated by 3-mercaptopyruvate sulfurtransferase (3-MST, EC 2.8.1.2) to form H_2_S (Fig. 3) (45). More recently, a new pathway to generate H_2_S using d-cysteine has been identified. The enzymes d-amino acid oxidase (DAO) along with 3-MST carry out biogenesis of H_2_S from d-cysteine (49). Nonenzymatic reactions also lead to the generation of H_2_S inside cells, however, their contribution is minor and remain poorly understood and characterized.

### Cystathionine Beta Synthase

Human cystathionine beta synthase (CBS) is a tetramer and is allosterically stimulated by S-adenosyl methionine (SAM) which binds to a conserved “CBS pair domain” in the C-terminal end of the protein (50,51). CBS catalyses the first and committed step of the transsulfuration pathway which canonically, leads to the production of cystathionine from serine and homocysteine (52). However, when serine is replaced by cysteine, H_2_S is produced (Fig. 3). CBS can generate H_2_S by additional reactions including *β* replacement of cysteine by water to form serine and *β* replacement of cysteine by a second molecule of cysteine to form lanthionine. In kinetic terms, the *β* replacement of cysteine with homocysteine remains the most favorable (52). As mentioned earlier, CBS utilizes PLP as a cofactor and is a type-II PLP binding protein (53). The cofactor remains covalently linked to the active site lysine via Schiff base formation and is pertinent to the enzymatic activity of the protein. In addition to PLP, human CBS also contains heme which acts as a redox dependent gas sensor (54). Apart from this the heme moiety also functions as a “metabolic switch” committing the pathway toward H_2_S production (55). Under ER stress, heme oxygenase is induced, which catabolises heme in presence of molecular oxygen to produce biliverdin and CO, the later one binds to heme cofactor of CBS and inhibits its activity. This inhibition leads to low levels of cystathionine and increased levels of homocysteine. These metabolites cue the second enzyme, CSE, to increase the production of H_2_S from cysteine and homocysteine further highlighting the metabolic flexibility of this pathway (55).

### Cystathionine Gamma Lyase

As the name suggests, the second enzyme of the pathway CSE, primarily catalyses the cleavage of cystathionine to form cysteine, ammonia and *α*-ketobutyrate (52). Human CSE is a homotetramer and a PLP binding protein. It can catalyse the production of H_2_S from cysteine and homocysteine alone or in combination (Fig. 3). The substrate promiscuity of CSE permits the accommodation of cysteine, homocysteine, and cystathionine in the same binding pocket. Regulation of CSE is not very well known but as mentioned earlier, on ER stress it upregulates the production of H_2_S by utilizing cysteine and homocysteine over cystathionine as substrates (55).

#### Mercaptopyruvate Sulfurtransferase

3-mercaptopyruvate is generated by a transamination reaction between cysteine and *α*-ketoglutarate catalysed by aspartate/cysteine aminotransferase (56). This 3 mercaptopyruvate (3-MP) is subsequently used as a substrate by 3-MST to form H_2_S (Fig. 3). 3-MST transfers the sulfur to a nucleophilic cysteine in the active site. This leads to the formation of a bound persulfide which acts as a source of H_2_S under reducing conditions or in presence of acceptors like thioredoxin. 3-MST is localized to the mitochondria unlike CBS and CSE which are cytosolic, where it is believed to contribute bioenergetically via sulfide oxidation. Also, unlike the transsulfuration enzymes, 3-MST is inhibited under oxidizing conditions due to a labile active site cysteine which gets converted to cysteine sulfonate leading to enzyme inactivation (57).

#### MST/DAO Pathway

In addition to l-cysteine, H_2_S production was observed in brain homogenates when d-cysteine was used as a substrate. This led to the discovery of new pathway involving peroxisomal enzyme d-amino oxidase (DAO) in H_2_S biogenesis. d-cysteine is metabolized by DAO to 3-mercaptopyruvate (3MP), which then translocates to mitochondria where it is converted to H_2_S and pyruvate. It has been reported that the production of H_2_S from d-cysteine is ~ 60 times greater in comparison to l-cysteine. Since DAO is only localized to the brain and the kidney, the functionality of the 3MST/DAO pathway for the production of H_2_S is only relevant to these tissues (49,58).

## Chemical Biology of H_2_S

H_2_S gas was discovered in 1777, by Carl Wilhelm Scheele, and was largely considered as a toxic gas for over hundred years (59). Based on toxicological studies, the permissible exposure limit of H_2_S is 10 ppm and 800 ppm exposure for 5 min is the lethal concentration for 50% of humans (LC50) (60,61). Much of its toxicity is owed to the fact that H_2_S is known to inhibit respiration thereby acting as a metabolic poison. When present at higher concentrations it causes reversible inhibition of cytochrome c oxidase (complex IV), thereby perturbing mitochondrial respiration and oxidative phosphorylation (46). This is further exemplified by the observations that H_2_S induces a state of “suspended animation” with consequent lowering of metabolic rate and body temperature in mice (61).

The standard two electron redox potential of H_2_S/S^0^ couple, −0.23 V (noted +0.140 value in acidic condition) at pH 7 (versus the standard hydrogen electrode) is comparable to that of major cellular antioxidant buffers, glutathione disulfide/glutathione (*E*°′= −0.262 V) and cystine/cysteine (*E*°′= −0.245 V) redox potentials (62–65). While the physiological concentration of H_2_S is a matter of ongoing controversy, it seems that low nM concentrations are most likely (66). Only in case of aorta, the reported concentration of free H_2_S is ~ 20–100 fold higher than that of other tissues (67). Interestingly, the flux of sulfur into H_2_S is comparable to that of GSH, indicating that the low nM levels are maintained as a consequence of higher sulfide clearance rate (59,68). The low steady-state concentration of H_2_S than GSH (~10 mM) precludes its involvement in counteracting oxidative stress by acting as an antioxidant buffer (46). Alternatively, it is proposed that H_2_S can modulate intracellular redox signaling by modifying cysteine thiols of the various cellular proteins (S-persulfidation) coordinating redox homeostasis. However, since a direct reaction of H_2_S with thiols is unlikely, the mechanism by which persulfides are formed intracellularly is poorly understood (69,70).

H_2_S is lipophilic and is known to permeate freely through biological membranes without any assistance from membrane channels (lipid bilayer permeability *P_M_* ≥ 0.5 ± 0.4 cm/s) (71). Being a weak acid, it dissociates immediately and equilibrates with its anion HS^–^ and S^2–^ in aqueous solution as shown in Eq. (1).

(1) $${\text{H}}_{2}\text{S}\overset{{\text{K}}_{1}}{⇌}{\text{H}}^{+}+{\text{HS}}^{-}\overset{{\text{K}}_{2}}{⇌}2{\text{H}}^{+}+{\text{S}}^{2-}$$

The p*K*_a1_ for H_2_S dissociation ranges from 6.97 to 7.06 at 25 °C, while p*K*_a2_ is estimated to be between 12.20 to 15.00 at 25 °C (72). Based on these values it is calculated that the ratio of HS^–^:H_2_S is 3:1 at physiological pH of 7.4 (72). Nevertheless, total intracellular H_2_S levels is referred to as total free sulfide pool (i.e., H_2_S + HS^–^ + S^2–^). Based on its chemical features, H_2_S can influence cellular redox physiology via four mechanisms: (1) scavenging of ROS and RNS, (2) reaction with metal centres, (3) modulation of cellular respiration, and (4) reaction with protein cysteine thiols to generate persulfides (S-persulfidation- an oxidative posttranslational modification [oxPTM]) (69,73). These mechanisms are described in the following section.

### H_2_S as a Free Radical Scavenger

H_2_S acts as a cytoprotective molecule and has the ability to directly scavenge free radical species (74). Owing to its nucleophilic properties, H_2_S has been shown to react with oxygen (O_2_), ROS, peroxynitrite (ONOOH/ONOO^–^), and hypochlorite (HOCL/^–^OCL) (65,75). The apparent second-order rate constants of H_2_S with various oxidants have been summarized in Table 1. While these studies indicate a direct scavenging of oxidants by H_2_S *in vitro*, the low concentrations of H_2_S (10 nM to 3 *μ*M) compared to other antioxidants (GSH; 1–10 mM) *in vivo* raised substantial concerns about its role in remediating ROS/RNS under biologically relevant conditions (66,76–79). Alternatively, H_2_S has been shown to increase GSH production by enhancing the inward transport of cystine and inducing the expression of GSH-biosynthetic enzyme, GCL (*γ*-GCS) (80,81). This increase in intracellular GSH could be another mechanism by which H_2_S indirectly participates in protection from oxidative stress.

### Reaction with Metal Centers

The interaction of H_2_S with metals falls into two categories: (i) electron-transfer reaction and (ii) coordinate complex formation (65). In the first category, complete electron transfer occurs between the sulfide species and the metal, whereas coordinate complex formation involves binding of the sulfur species to the metal ligand (65). These reactions are predicted on the basis of chemical properties of H_2_S to act as a nucleophile. Interestingly, a wine-like model was used to study the reaction mechanism of metals with H_2_S. Sulfidic off-odors encountered during wine production are due to the presence of H_2_S and low-molecular-weight thiols (82). These off-odors are usually removed in a process called Cu fining, wherein Cu (II) is added to selectively and rapidly form ~1.4:1 H_2_S/Cu and ~2:1 thiol/Cu complexes, resulting in oxidation of H_2_S and reduction of Cu (II) to Cu (I) (82). The CuS precipitate formed is than subsequently removed from the wine by racking and/or filtration (82).

In a biological setup, interaction of H_2_S with the mitochondrial heme protein-cytochrome C oxidase (CcO) is extensively studied. It has been demonstrated that high concentrations of H_2_S competitively binds to CcO, resulting in inhibition of O_2_ binding (83–85). H_2_S interacts with CcO through the O_2_-binding copper (Cu_B_)/heme (a_3_) iron binuclear site in oxidized state (Cu^2+^/Fe^3+^) and reduces the enzyme (86). The K_i_ for this reaction is 0.2 μM with purified CcO (86). Most of the studies demonstrating inhibitory effect of H_2_S on respiration via interaction with CcO were done using very high/nonphysiological concentrations of H_2_S. However, it was shown that the liver mitochondria of H_2_S treated rats show biphasic respiration profile (87). Low concentrations (0.1–3.0 μM) of H_2_S induces respiration whereas higher concentrations (30–100 μM) inhibits (87). At lower concentrations, H_2_S acts as a mitochondrial electron donor and stimulates electron transport chain (87,88).

Other than CcO, H_2_S is known to covalently modify ferryl/peroxo heme within hemoglobin and myoglobin resulting in the formation of green colored sulfhemoglobin and sulfmyoglobin species, both of which are indicators of H_2_S poisoning (89). Additionally, H_2_S can react with nonheme iron present in iron-sulfur cluster containing proteins to generate insoluble precipitates (65). Lastly, H_2_S is reported to react with a copper-containing protein (Cu—Zn—SOD) (90). This reaction involves copper-catalysed reduction of ${\text{O}}_{2}^{-}$ to H_2_O_2_ Oxidation of H_2_C to S^0^ (90).

### H_2_S and Cellular Bioenergetics

The effects of H_2_S on cellular bioenergetics are largely derived from examining mitochondrial function. The effect of H_2_S on mitochondria is complex, exhibiting two opposing effects; inhibition and stimulation of mitochondrial bioenergetics (88). Oxidation of H_2_S by mitochondrial inner membrane localized Sulfide-Quinone oxidoreductase (SQR) leads to transfer of electron from H_2_S to ubiquinone and increases the flux of electron transport to mitochondrial respiratory complex III and IV, thereby leading to enhanced oxygen consumption and cellular respiration (91,92).

Recently, it was demonstrated that H_2_S has a biphasic effect on cellular oxygen consumption/mitochondrial electron transport (87,88). These investigators creatively adapted Seahorse XF technology to precisely measure dynamic changes in mitochondrial bioenergetics in real-time in response to a gradient of H_2_S. Interestingly, treatment of isolated mitochondria with low H_2_S concentrations, NaHS (<1 μM) enhances mitochondrial oxygen consumption rate (OCR), ATP turnover rate and leads to increased maximal respiratory capacity (87). In contrast, treatment with high concentrations of NaHS (30–300 μM) causes reduced mitochondrial OCR and ATP generation, which is consistent with previous studies showing the inhibitory effect of H_2_S on mitochondrial respiration when present at high concentrations (84,93). The low endogenous concentrations of H_2_S in mitochondria is primarily maintained by mitochondrial localized 3-Mercaptopyruvate sulfurtransferase (3-MST) (87). Supplementation of isolated mitochondria with 3-mercaptopyruvate (3-MP) leads to enhanced H_2_S production by 3-MST pathway and induces mitochondrial bioenergetic parameters (87). Subsequently, genetic silencing of 3-MST leads to reduced basal OCR and due to absence of 3-MST there is no stimulatory effect of 3-MP on mitochondrial bioenergetic parameters (87).

Furthermore, it was found that the stimulatory effect of 3-MP on mitochondrial bioenergetic parameters was absent in mitochondria isolated from aged mice as compared to young mice (88). Moreover, exposure of H_2_S was shown to cause physiological alterations which enhanced thermotolerance and life span of *Caenorhabditis elegans* (94). The other two PLP-dependent cytosolic enzymes CBS and CSE also maintain endogenous H_2_S levels. Under stress condition, CSE is known to translocate into mitochondria and stimulate mitochondrial H_2_S generation. This subsequently results in an increased ATP generation and resistance to hypoxia (95). Furthermore, elevated endogenous H_2_S in colon cancer cells have been shown to regulate cell migration and invasion (96). Additionally, pharmacological inhibition of H_2_S production diminished the growth of cancer cells by suppressing basal respiration, ATP production, spare respiratory capacity, and glycolysis (96,97).

### Protein Persulfidation as a Mechanism of H_2_S Mediated Signaling

**Fig 4:**
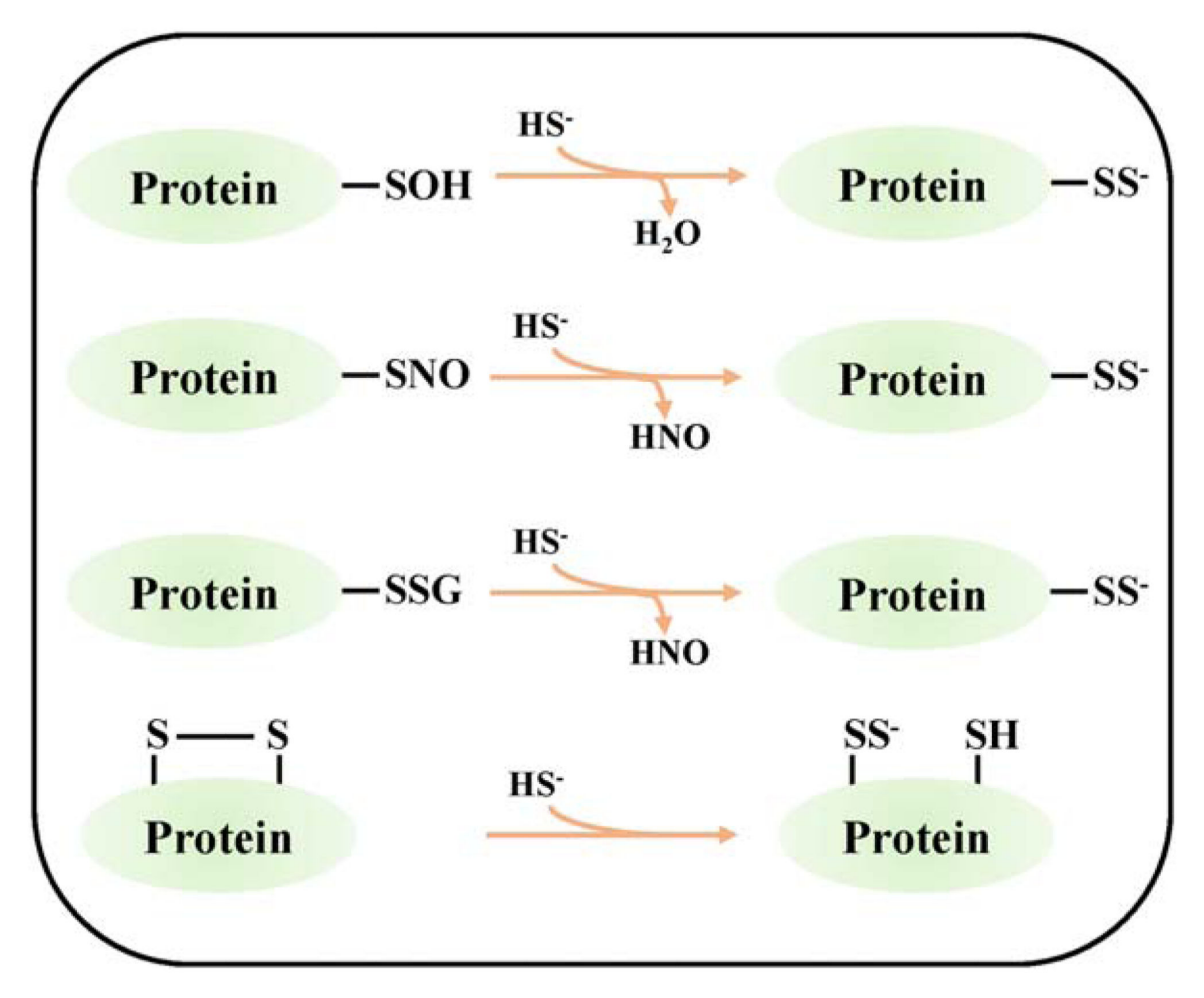
Proposed reaction mechanisms for H_2_S mediated S-persulfidation of proteins. From Top: H_2_S reacts with sulfenylated (—SOH), S-nitrosylated (—SNO), S-glutathionylated (—SSG) and disulfide form of cysteine in proteins by S-persulfidation.

H_2_S exerts its signaling property via oxidative posttranslational modification (oxPTM) of cysteine residues, called S-persulfidation (98–100). Persulfidation (R-SSH) is the oxidation of thiol (—SH) from −2 to −1 oxidation state (101). Being a reductant, H_2_S/HS^–^ can carry out the persulfidation reaction only when one of the reagents (—SH group of proteins or H_2_S) is in the oxidized form (101). The proposed reactions for the formation of S-persulfide bond by the nucleophilic attack of HS^–^ anion on reversibly oxidized protein —SH group is shown in (Fig. 4).

Recently, it was reported that persulfidation protects proteins from oxidative stress-induced damage and the over oxidized persulfidated cysteine sulfonic acid $\left(\text{P}—\text{SS}{\text{O}}_{3}^{2-}\right)$ can be reversed to thiol (—SH) by the depersulfidase activity of thioredoxin (102). Thus, persulfidation can act as a protective mechanism against oxidative stress-induced protein damage (102). Using LC–MS-based techniques, S-persulfidation of proteins involved in fatty acids and carbohydrate metabolism, cellular response to stress, cell redox homeostasis, translation, and cell cycle were identified (73). Similarly, H_2_S was shown to modify cysteines in about 10–25% of liver proteins including actin, tubulin, and glyceraldehyde-3-phosphate (GAPDH) by persulfidation under physiological conditions and modulate their activities (103). Several key host transcription factors, Nrf2 and Nf-*κ*B, are directly targeted by H_2_S via S-persulfidation (98). Apart from eukaryotic systems, a recent study, for the first time, assessed widespread S-persulfidation of proteins in *Staphylococcus aureus* (*S. aureus*) (104). More importantly, H_2_S exposure increased the level of S-persulfidation, whereas mutations in transsulfuration pathways (*cysM* and *metB*) had an opposite effect (104). Lastly, this study revealed extensive persulfidation of transcription factors involved in virulence regulation (SarA family) and interaction with host immune response (superantigen-like proteins [SSLs]) (104).

## Detection of H_2_S and Protein S-Persulfidation

### Tools for Detection and Quantitation of H_2_S

To achieve a better understanding of the physiological effects of H_2_S, it is imperative to determine the levels of this gasotransmitter in its free and other biological forms. This becomes especially important as the role of H_2_S as a protectant versus poison has been contended throughout the course of its study as a biological effector molecule. The suitability of any detection method relies heavily on three pertinent aspects namely sensitivity, reproducibility and experimental ease. A multitude of detection methodologies are available for qualitative and quantitative measurement of H_2_S levels. While this may seem advantageous at first glance, it has led to a huge variability in the reported levels of “bioavailable” H_2_S throughout literature. Furthermore, additional factors influencing detection arise due to experimental aspects like biological sample (tissue, sera, and cells), pH, and oxygen. The latter have been demonstrated to exert a direct effect on the stability of H_2_S (105).

The detection methods range from simple colorimetric assays to techniques like gas chromatography (GC), High pressure liquid chromatography (HPLC), electrochemical, polarographic, fluorescent, and recently developed nanotechnology-based systems. Some of these techniques are summarized in Table 2. Although these methods have been widely used for biological H_2_S detection, they are not devoid of pitfalls. The issues of sensitivity, invasiveness, artefactual readout, half-life, stability, permeability, lack of spatio-temporal insight, and cumbersome experimental setup cannot be overlooked. Therefore, the reporting and interpretation of experimental data becomes immensely influenced by the method that a researcher chooses to adopt. Many authors have argued that for a dynamic gaseous effector molecule like H_2_S, the absolute numbers in terms of concentration may actually not matter as much as the determination of the qualitative trend of its rise and fall under different physiological conditions. This may, however, not hold true for pathophysiological conditions wherein accurate determination of H_2_S levels may become indispensable for diagnosis of certain diseases.

Apart from the aforementioned techniques, recent studies using H_2_S sensitive fluorescent proteins as genetically encoded biosensors have garnered immense interest. GFP molecule has been reengineered to contain the unnatural amino acid *p*-azidophenylalanine (pAzF). This azido group can be reduced specifically by H_2_S imparting selectivity to the GFP molecule, now termed as hsGFP. An orthogonal tRNA-tRNA synthetase system from *E. coli* was used for the selective incorporation of pAzF into hsGFP molecule in response to the Amber (TAG) codon. In presence of both H_2_S and pAzF, the chromophore *p*-azidoben-zylideneimidazolidone of hsGFP is converted to *p*-aminobenzy-lideneimidazolidone with fluorescence excitation and emission maxima at 454 nm and 500 nm, respectively. This genetically encoded probe has been used in mammalian cell line HEK293T for the detection of intracellular H_2_S. Such a genetically encoded biosensor is noninvasive and can reflect the dynamic nature of the turnover of H_2_S inside cells (128). Furthermore, such sensors can be targeted to specific cellular locations. Taken together, a genetically encoded biosensor appears to be a promising tool to study the levels of H_2_S under physiological conditions.

### Tools for Detection of Protein S-Persulfidation

In addition to H_2_S, the detection of the post translational modification caused by it is also of the utmost importance to fully appreciate the functional relevance of this gasotransmitter. Protein persulfidation was identified to be the mechanism by which H_2_S exerts its signaling function (103). Detection of this modification, however, poses a significant challenge as the persulfide group exhibits similar reactivity to free thiols (129). Table 3 summarizes some of the widely used methods for the detection of intracellular protein persulfidation levels.

## Role of H_2_S in Bacterial Physiology

While the initial co-culture experiments described earlier provided a valuable clue with regard to potential of H_2_S in protecting bacteria from toxic compounds, in depth examination of these findings was never attempted (42,43). It was only in 2011 that a study highlighted the importance of H_2_S in protecting bacteria from antibiotics and oxidative stress (44). In this context, H_2_S has been termed as a “double edged sword” mitigating not only the effects of antibiotics but also the resulting oxidative stress caused by them. To ascertain the role of H_2_S in *E coli*, the authors compared wild type and 3-MST deficient *E. coli* by a phenotypic microarray. While these strains showed no difference with respect to growth defects *in vitro*, the 3-MST deficient strain became highly susceptible to structurally and functionally different classes of antibiotics. Similar results were obtained for CBS/CSE deficient strains of *P. aeruginosa*, *S. aureus*, and *B. anthracis*, establishing the protective role of H_2_S across gram negative and gram-positive bacteria. Overexpression of 3-MST led to enhanced protection against spectinomycin whereas chemical inhibition on 3-MST, CBS, and CSE rendered them highly susceptible to a variety of antibiotics. NaHS, an H_2_S donor chemically complemented these mutant strains establishing the role of endogenously generated H_2_S as a protective mechanism against antibiotics.

**Fig 5:**
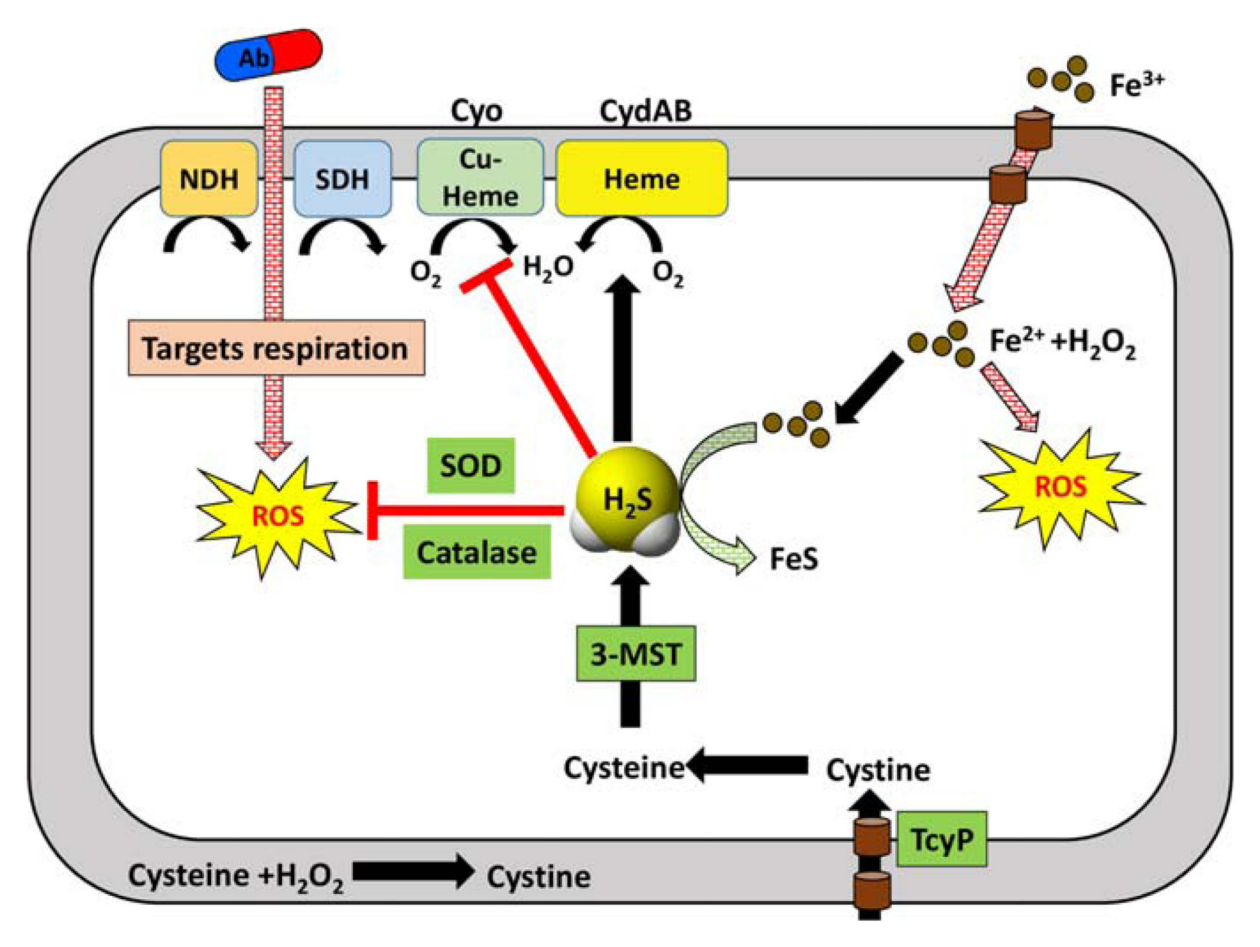
Hydrogen sulfide and its cytoprotective effect in *E. coli*. H_2_S produced by 3-MST can directly sequester Fe^2+^ ions thereby preventing the detrimental Fenton reaction which lead to the production of ROS. H_2_S promotes tolerance to antibiotics by upregulation of cytochrome bd oxidase (CydAB) and downregulating cytochrome bo oxidase (Cyo) to maintain respiratory flux and redox poise. Additionally, H_2_S augments catalase and super oxide dismutase (SOD) activities. Abbreviations: NDH, NADH dehydrogenase; SDH, succinate dehydrogenase; Ab, antibiotics; TcyP, Transporter of l-cystine.

Several studies have shown that a wide range of antibiotics exert killing by triggering ROS generation in addition to inhibiting the function of their primary targets (135,136). Antibiotics have been shown to stimulate respiration, which increases generation of toxic hydroxyl radicals via Fe^2+^-catalysed Fenton reaction (137). Consistent with these observations the authors have shown that pretreatment with Fe-chelator (dipyridyl) or ROS scavenger (thiourea) induces gentamycin resistance to both the wild type and H_2_S deficient strains of *E. coli*. Interestingly, a similar degree of protection from gentamycin was observed when the cells were treated with NaHS. Additionally, all the H_2_S deficient mutant strains exhibited severe susceptibility to H_2_O_2_ which was mitigated when they were pretreated with NaHS. DNA damage is one of the direct consequences of oxidative stress generated by antibiotics (138,139). On treatment with sublethal levels of ampicillin, which is known to cause oxidative stress, 3-MST deficient strain of *E. coli* showed tell-tale signs of DNA damage. Overexpression of 3-MST and pretreatment with NaHS ameliorated this damage. Additionally, the antioxidant effect of H_2_S was also shown to be in part due to the stimulation of antioxidant enzymes such as catalase and superoxide dismutase (SOD). Consistent with this, the rate of degradation of H_2_O_2_ was significantly greater in the crude cell lysates of wild type as compared to 3-MST deficient *E. coli*. However, the precise biochemical mechanism by which H_2_S modulates the activity of antioxidants was left unaddressed. Recently, direct sequestration of Fe^2+^ ions by H_2_S has been shown to counteract antibiotics triggered oxidative stress (140). This mechanism became very apparent in *E. coli* strain deficient in an iron uptake regulator, Fur. The *fur* mutant cells constitutively take up iron resulting in iron accumulation and consequent DNA damage and killing by H_2_O_2_. Interestingly, susceptibility to ROS is further exacerbated in the *fur/3-mst* double mutant, whereas overexpression of *3-mst* in Fur deficient cells completely reversed both DNA damage and killing. All of these findings indicate that H_2_S can mitigate oxidative stress by potentiating the activity of antioxidant enzymes and sequestering Fe^2+^ (Fig. 5).

Apart from the above studies, another mechanism was put forward to explain the protective role of H_2_S. This involved the master regulator CysB which is involved in regulation of a number of sulfur metabolism genes in *E. coli*. CysB regulates the expression of TcyP, a cystine importer. During oxidative stress, H_2_O_2_ interacts with cysteine leading to its depletion and induction of CysB regulon including TcyP. As a consequence, TcyP leads to an increased influx of cystine/cysteine inside the cytoplasm resulting in the enhanced production of H_2_S via 3-MST. More recently, using chemical-biology approaches, we developed a series of bacteria specific H_2_S donors to explain the mechanism of H_2_S mediated protection (141). On pretreatment of *E. coli* with one such H_2_S donor (1c), we showed increased resistance to bactericidal antibiotics (ampicillin and amikacin) and H_2_O_2_. Furthermore, using a noninvasive biosensor of cytoplasmic redox potential (roGFP2) (142,143), we for the first time, precisely measured real-time changes in the redox physiology of *E.coli* in response to antibiotics in the presence or absence of H_2_S. Importantly, we showed that while elevation in endogenous H_2_S levels does not influence redox physiology of *E.coli*, it efficiently reversed antibiotics induced oxidative shift in the cytoplasmic redox potential of bacteria. To further probe mechanistic aspects of these findings, we discovered a functional association between H_2_S-directed cytoprotection and alternate mode of cellular respiration catalysed by cytochrome bd oxidase (CydB). H_2_S, due to its strong affinity for metals such as copper, is known to inhibit copper-heme containing cytochrome bo oxidase (CyoA). Under these conditions, the respiration proceeds via a less energy efficient CydB. In agreement with this, treatment of the cells with 1c led to a downregulation of *cyoA* transcript, whereas the transcripts of alternate respiratory oxidases such as *cydB* and *appY* were either maintained or enhanced, respectively. This realignment of respiratory oxidases mimics the expression profile of *E. coli* grown under respiratory arrest conditions (e.g., hypoxia), implicating H_2_S in respiration inhibition and metabolic slow down. In contrast, ampicillin treatment enhanced the expression of the energy efficient *cyoA* and repressed *cydB*, consistent with the reported hyperactivation of electron transport chain by bactericidal antibiotics (144). However, pretreatment of the cells with 1c reversed the influence of ampicillin on *cyoA* and c*ydB* transcripts. In agreement with these findings, the *cyoA* mutant pretreated with 1c remained protected from ampicillin toxicity, whereas 1c-derived H_2_S remained completely ineffective in protecting *cydB* mutant. The *cydB* from *E. coli* has also been shown to reduce H_2_O_2_ by acting as catalase and quinol peroxidase (145). Therefore, sustenance of *cydB* expression by H_2_S can potentiate antibiotic tolerance by bolstering the bacterial antioxidant capacity. Altogether, these observations put forth a central role of alternate respiration and oxidant mitigating mechanisms in resisting the activity of antibiotics by H_2_S.

The association of H_2_S and drug resistance is not new. Nearly 40 years ago, several studies have reported the presence of plasmid-borne genetic elements enhancing both basal H_2_S production and antibiotic resistance in multidrug-resistant strains of *E. coli* isolated from patients suffering from urinary tract infection (146,147). Recently, we confirmed these findings and demonstrated that clinical strains of multidrug resistant uropathogenic *E. coli* possesses greater levels of endogenous H_2_S as compared to wild-type. Intriguingly, pretreating these strains with aspartate, a 3-MST inhibitor significantly reduced endogenous H_2_S levels and markedly reversed (50%) resistance toward ampicillin. More importantly, exposure to H_2_S donor neutralized the adverse effect of aspartate on drug resistance. Our work provided a strong pharmacological foundation for design of inhibitors of H_2_S biogenesis as a possible adjuvant to antibiotics. Further, it appears that targeting antioxidant enzymes and alternate respiratory complexes (Cyd/App) is likely to potentiate the killing efficiency of antibiotics.

The effect of H_2_S on intracellular human pathogens such as *Mycobacterium tuberculosis* (*Mtb*) is largely unknown. However, a recent study aimed at identifying genetic components involved in protection from oxidative stress showed that H_2_S donor (NaHS) can complement the defects in recycling of the major mycobacterial antioxidant, mycothiol (MSH) (148). Using a specific biosensor of mycothiol redox potential (*E*_MSH_; Mrx1-roGFP2) (143), authors have shown that NaHS restored steady-state MSH/MSSM ratio (basal *E*_MSH_) in the mutants showing oxidative *E*_MSH_. It is likely that NaHS increased mycothiol biosynthesis similar to H_2_S-mediated increased biogenesis of GSH in eukaryotes. This supplementation with NaHS also enhanced the survival of the *Mtb* mycothiol recycling mutants in both activated macrophages and animals. Thus, the contribution of endogenous H_2_S biogenesis pathways in regulating redox balance, stress response, and virulence is eagerly awaited in several human pathogens including *Mtb*.

## Oxidative Stress is an Integral Part of Infection with Diverse Class of Viruses

Oxidative stress has been linked to vast group of etiological agents that cause acute and chronic diseases such as infection with viruses, bacteria, and parasites (149). Viral and bacterial infections in particular have been linked to induce ROS/RNS production, alteration in metabolic pathways, and leading to several disease associated complications (149,150). However, this field still lacks mechanistic insight. The impact of these infectious agents on host redox physiology and how this could be targeted for therapeutic benefits remains a challenging area of research.

In case of viral infections, induction of oxidative stress inside host is a prerequisite for successful infection and long term viral replication. RNA viruses such as influenza and paramyxovirus infection generates ROI via activation of monocytes and polymorphonuclear leukocytes (149). A study indicates that the oxidative state of the host cells provides an environment permissive for viral replication (151). Using mice models, it has been shown that influenza A (RNA virus) infection creates redox imbalance by decreasing the levels of GSH, vitamins C, and vitamin E (152). Similarly, a transgenic mouse model of hepatitis B virus (DNA virus) was exploited to show that this virus induces ROS and leads to hepatocarcinogeneis and oxidative DNA damage during chronic necroinflammatory conditions (153). Based on these studies, it has been proposed that antioxidant strategies can be utilized to target viral replication and to decrease viral induced oxidative stress to control pathological manifestations (154).

**Fig 6:**
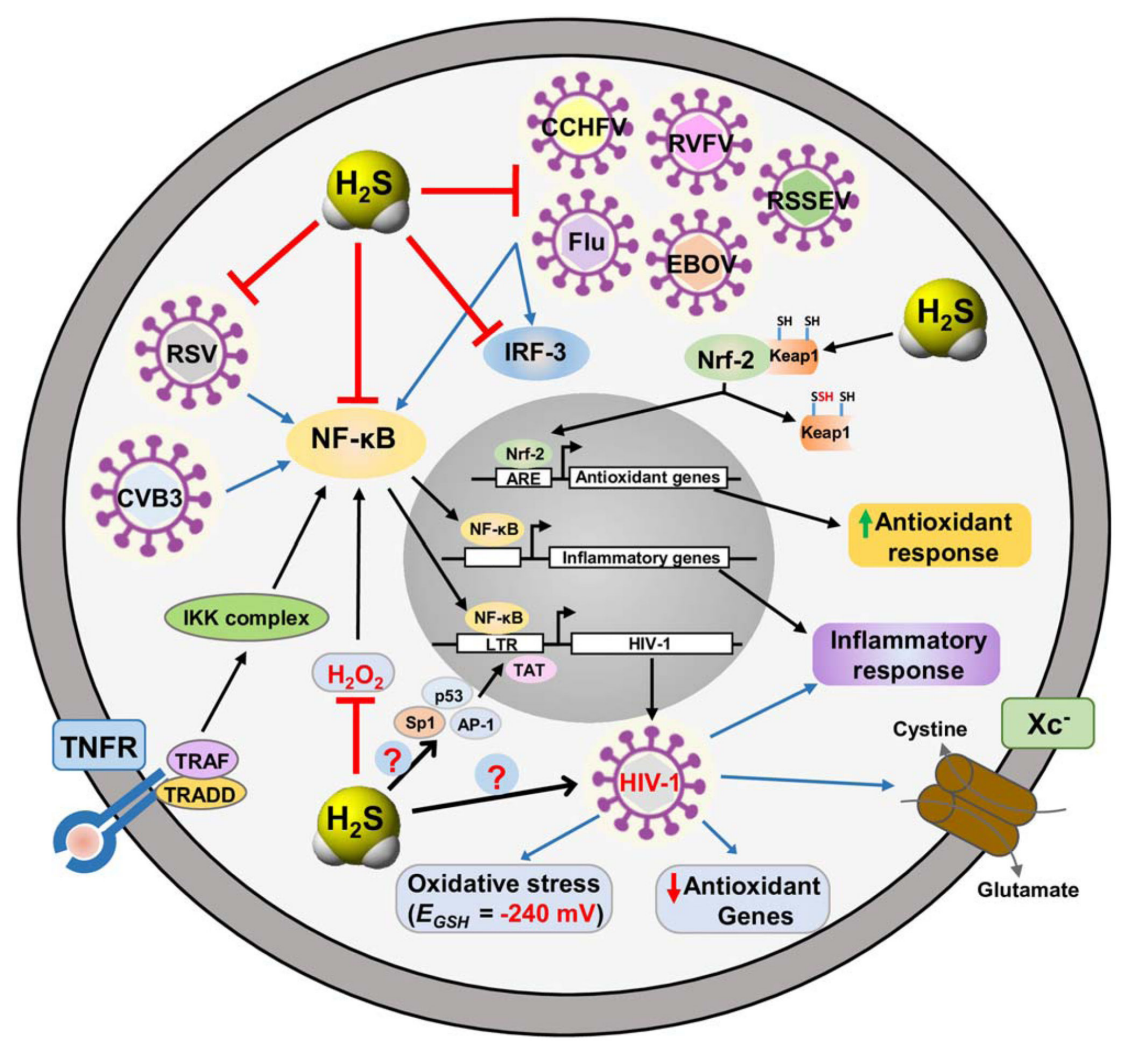
H_2_S targets key host factors and viral steps to perturb replication of enveloped RNA viruses. H_2_S acts as an antioxidant gaseous signaling molecule by directly scavenging ROS and upregulating cellular antioxidant capacity via Nrf-2/ARE pathway. Moreover, H_2_S has been shown to exert broad range antiviral and anti-inflammatory activity. Redox sensitive transcription factors Nf-*κ*B, Ap-1, p53, and Sp1 mediates HIV-1 reactivation from latency in conjunction with viral Trans-activator of transcription (Tat). As pathways related to redox metabolism are significantly affected on HIV-1 replication it would be of great interest to characterize the role H_2_S in HIV-1 latency and reactivation. Abbreviations: RSV, Respiratory Syncytial Virus; CVB3, Coxsackie virus B3; Flu, Influenza virus (H1N1 (A strain), H3N2 (A strain) and Brisbane (B strain)); EBOV, Ebola virus; RVFV, Rift valley fever virus; CCHFV, Crimean-Congo hemorrhagic fever virus; RSSEV, Far-eastern subtype tick-borne flavivirus; Nf-*κ*B, Nuclear factor kappa-light-chain-enhancer of activated B cells; IRF-3, Interferon regulatory factor 3; TNFR, tumor necrosis factor receptor; Xc-, cystine/glutamate transporter; TRADD, Tumor necrosis factor receptor type 1-associated DEATH domain protein; TRAF, TNF receptor associated factor.

The role of oxidative stress has been extensively studied in retroviruses such as HIV-1 (Human Immunodeficiency Virus). Studies have shown that HIV-1 replication induces ROS generation and decreases cellular antioxidants such as GSH and Trx, and modulates immunopathogenesis during AIDS progression (155,156). Plasma and peripheral blood mononuclear cells (PBMCs) of AIDS patients show reduction in concentration of other major antioxidants like cysteine, methionine, vitamins C and E, along with elevated levels of lipid peroxidation products (157). At molecular level, ROS has been shown to induce the activity of redox sensitive transcription factors Nf-*κ*B, AP-1, and Sp1, which regulates HIV-1 gene transcription by binding to 5′-LTR promoter (158,159). Despite these studies, for a very long time, the relation between HIV-1 and oxidative stress remained circumstantial. This was largely owing to the lack of sophisticated and sensitive tools to measure intracellular redox potential of HIV-1 infected cells during various stages of infection. To fill this knowledge gap, we exploited a noninvasive biosensor of GSH redox potential (Grx1-roGFP2; *E*_GSH_) and accurately measured oxidative stress in the cytoplasm and mitochondria of HIV-1 infected monocytes (160). We demonstrated that monocytes latently infected with HIV-1 are intrinsically resistant to oxidative stress and displayed reductive *E*_GSH_ (160). More importantly, we showed that a marginal oxidative shift in *E*_GSH_ (25 mV) triggers reactivation of HIV-1 without adversely affecting cellular physiology. Furthermore, supplementation with antioxidants such as *N*-acetylcysteine kept HIV-1 in a silent state by preventing an oxidative shift in *E*_GSH_ required for viral activation (160). Lastly, global expression analysis revealed that pathways associated with redox metabolism are significantly affected during HIV-1 latency and reactivation (Fig. 6) (160). Taken together, these results highlight the central role of host redox physiology in modulating HIV-1 life cycle.

At molecular level HIV-1 infection or exposure to HIV-1 related proteins downregulates the Nuclear factor-erythroid-2 p45 related factor 2-Antioxidant Response Element (Nrf2/ARE) pathway, leading to reduced expression of antioxidant genes (161,162). Nrf2 is a constitutive transcription factor and master regulator of the antioxidant response (163,164). Nrf2 is inhibited in cytosol by Kelch-like ECH-associated protein-1 (Keap1), which is a redox-sensitive ubiquitin ligase substrate adaptor leading to ubiquitination and degradation of Nrf2 (165). Interestingly, Nrf2 inducer (Sulforaphane) has the ability to block HIV-1 infection in primary macrophages, which are the long-lived reservoirs of HIV-1 in infected individuals (166). In this direction, sulforaphane has also been shown to enhance phagocytic activity of HIV-1 infected monocytes-derived macrophages (MDMs) and alveolar macrophages (Ams) from HIV-1 transgenic rats, thereby reducing the severity of HIV-1 related pulmonary dysfunctions (161). Nrf2 activation has also been shown to assist Marburg virus ((-ss) RNA) (MARV), Kaposi’s sarcoma-associated herpesvirus (dsDNA) (KSHV), and Dengue virus ((_+_ss) RNA) replication. Activation of Nrf2 induced by VP24 and vFLIP proteins of MARV and KSHV, respectively, leads to dysregulation of host antiviral response and modulates viral gene expression, thereby ensuring a conducive environment for infection and also promotes the survival of infected cells (167,168). Dengue infection induced oxidative stress has been shown to activate Nrf2 thereby modulating the level of oxidative stress and affecting both antiviral and cell death response (169). While these studies clearly establish a connection between oxidative stress and infection with pathogenic viruses, the contribution of H_2_S in these process is poorly understood. Interestingly, Nrf2/Keap-1 pathway could provide the missing link between H_2_S, redox stress and virus infections. H_2_S has been shown to inhibit Keap1 by persulfidation of Cys 151, which leads to translocation of Nrf2 into the nucleus and its subsequent binding to Antioxidant Response Element (ARE). This results in the induction of genes encoding antioxidant and phase II detoxifying enzymes, such as heme oxygenase-1 (HO-1), NAD(P)H:quinone oxidoreductase-1 (NQO-1), glutamate cysteine ligase catalytic subunit (GCLC), and thioredoxin reductase-1 (TXNRD-1) (163,165). In relation to this H_2_S has been shown to protect against cellular senescence and oxidative stress via S-sulfhydration of Keap1 and resulting activation of Nrf2 (165). Further experimentation on H_2_S biogenesis and Nrf2/ARE pathway during viral infections will provide next stage of insight in this process. The following section provides recent knowledge in this direction.

## H_2_S and Host–Pathogen Interaction

Host-derived H_2_S has a marked effect on the outcome of bacterial and viral infections. Blocking the host transsulfuration pathway in macrophages by propargylglycine increased the viability of *Mycobacterium smegmatis*. This impairment in bacterial clearance was shown to be due to defects in the phagolysosomal fusion during infection. Treatment with *N*-acetylcysteine (NAC), which is known to increase cysteine flux through H_2_S biogenesis pathway, significantly increased the phagolysosomal fusion resulting in vacuolar acidification and killing of mycobacteria (170). H_2_S was found to inhibit the induction of an inflammatory response on infection with *Mycoplasma fermentans*. Underlying mechanism revealed that H_2_S inhibited the activation and nuclear translocation of a redox sensitive transcription factor NF-*κ*B, thereby diminishing the transcription of proinflammatory genes (171,172). One of the mechanism by which H_2_S affects the activity of a global transcriptional regulator, NF-*κ*B, is by persulfidation of Cys-38 residue in the p65 subunit (131).

H_2_S production by gut microbiota presents itself as an interesting line of study to explore the effect of bacteria-derived effector molecules affecting host physiology and pathophysiology. Sulfide reducing bacteria (SRB) represent a major class of the normal gut microbiota (173). The predominant genera residing in gut are *Desulfovibrio*, *Desulfobacter*, *Desulfolobus*, *and Desulfotomaculum*. SRBs are the major contributors of nonenzymatic H_2_S produced in the human body (174). Some of the initial studies to explore the significance of gut microbiota-derived H_2_S came from experiments done on germ free animals. It was observed that fecal samples of germ free mice contained half the H_2_S as compared to control mice (175). In addition to this, free H_2_S levels in inferior vena cava, blood plasma and in gastrointestinal tissues, were shown to be diminished in germ free mice. Apart from this, sulfane sulfur levels of plasma, adipose, and lung tissues were also found to be lower in such mice. This implicated the gut microflora as a potential source of circulating H_2_S for the host (176). Recent studies have also shown that colonocytes are capable of using H_2_S as an energy source (177).

Gut bacteria-derived H_2_S has been shown to have both protective and detrimental effects on colonic health. Increased fecal sulfide levels have been found in patients with ulcerative colitis (UC) (178). Furthermore, it has been suggested that epithelial damage associated with UC is due to increased availability of dietary sulfate for SRBs (179). In contrast, using animal models of colitis, it was demonstrated that scavenging bacterial H_2_S by bismuth did not ameliorate the symptoms of colitis (180). In fact, the condition was shown to improve on exogenous H_2_S administration (181). The ability of luminal H_2_S to modify secreted defensive proteins like trefoil factor 3 (TFF3) by reduction of the disulfide bond is believed to be a potential mechanism of the anti-inflammatory role of H_2_S. TFF3 plays an important role in mucosal repair and regeneration (182).

Our survey of literature revealed the existence of very few reports on H_2_S and viral infections. Existing studies have highlighted the role of H_2_S in modulating Respiratory Syncytial Virus (RSV) ((_-_ss) RNA virus) infection (183). RSV causes lower and upper respiratory tract infection in infants for which there is no vaccine and only limited supportive measures for treatment exist with no real benefits (184). RSV mediates its influence on the host by upregulating the expression of various Nf-*κ*B and IRF-3 dependent cytokines and chemokines (185). This leads to inflammation, cellular infiltration in lungs and other pulmonary dysfunctions. Importantly, RSV infection resulted in downregulation of expression and impaired activity of H_2_S biosynthesis enzymes. As a consequence, the endogenous levels of H_2_S were diminished in RSV infected cells (183). Pharmacological inhibition or genetic silencing of CSE (cystathionine gamma-lyase) enhances RSV multiplication and exacerbates disease condition, airway dysfunction, and pulmonary inflammation (186,187). Consistent with these findings, exogenous administration of H_2_S reduces the secretion of viral induced chemokines and cytokine through inhibition of NF-*κ*B mediated activation of genes encoding proinflammatory cytokines (183). H_2_S treatment (using slow releasing H_2_S donor-GYY4137) significantly blocked RSV replication *in vitro* and *in vivo* by targeting viral assembly, release, viral spread, and replication (Fig. 6) (183,186). Moreover, H_2_S treatment significantly improved clinical disease parameters and pulmonary dysfunction on RSV infection (186). Similarly, H_2_S also exerts antiviral and anti-inflammatory effects on the viruses in the family of *Paramyxoviridae*; human metapneumovirus (hMPV) and Nipah virus (NiV) (183).

H_2_S has also been shown to affect replication of highly pathogenic enveloped RNA virus from Ortho-, Filo-, Flavi-, and Bunyavirus families (Fig. 6) (188). FDA approved antiviral treatment is available for influenza virus (*Orthomyxoviridae*), whereas there is no vaccine or therapeutic interventions to target Ebola virus (*Filoviridae*), Far-eastern subtype tick-borne flavivirus (*Flaviviridae*), Rift valley fever virus and Crimean-Congo hemorrhagic fever virus (*Bunyaviridae*) (189). H_2_S was shown to significantly reduce replication of all the above families of viruses (188). As explained earlier, studies confirmed that H_2_S targets the transcription factor Nf-*κ*B and IRF-3 nuclear translocation to inhibit the release of viral induced proinflammatory mediators (188).

Lastly, H_2_S has been recently shown to modulate Coxsackie virus B3 (CVB3) infection induced inflammatory response, which is a predominant cause of human myocarditis and ultimately leads to heart failure (190). Treatment of CVB3 infected rats with H_2_S significantly resulted in downregulation of proinflammatory mediators, reduces myocardial injury, and alleviates damage of myocardial cells (191). H_2_S was shown to inhibit Nf-*κ*B signaling by lowering I*κ*B*α* degradation leading to reduced nuclear translocation and DNA binding ability of Nf-*κ*B (191). CVB3 infection also induces MAPK signaling cascade by activating ERK1/2, p38 and JNK1/2 which are upstream signaling molecule involved in activation of Nf-*κ*B (192). H_2_S treatment also showed reduced CVB3 induced activation of ERK1/2, p38, and JNK1/2 in rat myocardial cells, thereby supressing the expression of inflammatory mediators and alleviating myocardial damage (191).

## Conclusion

A bourgeoning body of literature stands testament to the cytoprotective role of H_2_S in various organs and tissues, ameliorating a wide variety of stresses. In comparison, the importance of this gasotransmitter from the perspective of bacterial physiology and pathogenesis remains understudied. This aspect becomes increasingly important as H_2_S has been shown to confer protection from both antibiotic and oxidative stresses. In today’s scenario, where drug resistance is becoming one of the leading healthcare concerns, there is an immediate need to have a greater understanding of the role of H_2_S in bacterial antibiotic resistance. Additionally, it would be worthwhile to study the importance of this molecule in intracellular bacteria, which face a hostile oxidative host environment.

Viral infections are accompanied by host physiological perturbations like alteration in redox homeostasis, inflammation, metabolism, among others. Of particular interest to us is the observation that a number of viruses have been shown to adversely affect the host H_2_S biogenesis. Antiviral and anti-inflammatory effects of H_2_S highlight its potential as a therapeutic molecule. It can, therefore, bolster the efficacy of the regular drug regime used for viral infections. Despite all these observations there is a dearth of knowledge in terms of molecular mechanism of these effects which could form a promising line of research. Through our review, we have highlighted some of the important studies in this field pertaining to the role of H_2_S in physiology and pathogenesis of these infectious agents. Considering that the role of H_2_S as a cytoprotectant appears to be conserved across phyla, the significance of this ubiquitous biological effector molecule in bacteria and viruses should be pursued with renewed interest.

## Figures and Tables

**Table 1:** Second-order rate constants of H_2_S with oxidants (Refs. 65,75)

Reaction with oxidants	Rate constants (M^−1^ s^−1^) at 37 °C

${\text{O}}_{2}^{-}$ + H_2_S/HS^−^ → H_2_O_2_ + S^−^	1.5 × 10^6^

HO^.^ + H_2_S/HS^−^ → S^−^ + H_2_O	1.5 × 10^10^

H_2_O_2_ + HS^−^ → HSOH + OH^−^	0.73

ONOOH + HS^−^ → HSOH + N${\text{O}}_{2}^{-}$	4.8 × 10^3^

HSOH + HS^−^ → HSSH + OH^−^	1.0 × 10^5^

HOCl + HS^−^ → HSCl + OH^−^	8.0 × 10^7^

S^−^ + S^−^ + 2H^+^ → HSSH	6.5 × 10^9^

**Table 2:** Tools for detection and quantification of H_2_S

Detection method	Description	References
Colorimetric	H_2_S reacts with metal salts like lead acetate, bismuth chloride, silver nitrate to form the lead sulfide which can be detected and quantified using UV-VIS spectroscopy.	(106–108)
	“Zinc trap” method in which zinc acetate reacts with H_2_S forming zinc sulfide with subsequent acidification with *N*, *N*-dimethyl phenylenediamine. The product can be detected and quantified using UV-VIS spectroscopy.	(109,110)
Chromatographic	Gas chromatography has been combined with flame photometric detectors, ion chromatography, silver particle trapping, and chemiluminescent detectors.	(20,67,111–114)
	HPLC of sulfide derivatized with monobromobimane, dibromobimane, *p*-phenylenediamine, and Fe^3+^. Reverse Phase HPLC of methylene blue formed by the zinc trap assay.	(115–118)
Electrochemical	Sulfide ion specific electrode measures S^2–^ form of sulfide which requires alkaline environment using Ag/Ag_2_S electrodes.	(119,120)
	Polarographic real time measurement of H_2_S using a polarographic oxygen sensor as anode and platinum wire as cathode and alkaline K_3_Fe(CN)_6_ as electrolyte. An H_2_S permeable membrane allows diffusion of H_2_S into the electrolyte solution reducing it. The electrolyte subsequently gets re-oxidized on the surface of the platinum electrode to produce a current proportional to H_2_S concentration.	(121,122)
Fluorescent sensors	All fluorescent probes consist of a fluorescence signal transducer and the fluorescence modulator. The transducer is a suitable fluorescent moiety while the modulator is chosen based on the chemical nature of H_2_S and physiologically permissible reaction kinetics. Fluorescent moieties like rhodamine, BODIPY, dansyl, 7-hydroxy-4-methylcoumarin, naphthilamide, cyanine, etc, have been used as transducers. The modulators have been designed based on the selective reduction of nitro groups to amines and azide groups to amines by H_2_S, thiolysis, addition and cyclization reactions based on the nucleophilic nature of H_2_S and copper sulfide precipitation resulting from the affinity of H_2_S for copper. Others include selenium-based probes for reversible detection of H_2_S. Probes have been designed based on the selenide-selenoxide redox reaction of many selenoenzymes. These probes can monitor redox cycling between H_2_S and ROS.	(123)
Nanotechnology-based sensors	Single walled carbon nanotube networks, gold nanoclusters, nanorods, nanocomposites of FAM DNA etched on the surface of silver nanoparticles have been employed for the detection of H_2_S.	(124–127)

**Table 3:** Tools for detection of protein S-persulfidation

Detection method	Description	References

**Biotin switch assay**	This was the first assay developed for the detection of persulfidation. It was based on the premise that persulfides would not react with electrophilic thiolblocking reagent S-methyl methanethiosulfonate (MMTS). Persulfides were subsequently labeled with *N*-[6-(biotinamido)hexyl]- 30 -(20 -pyridyldithio)propionamide (biotin-HPDP). The biotin labeled species were pulled down by streptavidin beads and analysed by MS. It was demonstrated that upto 25% of proteins in the liver were persulfidated under basal conditions. The chemistry was later on proved to be incorrect as persulfides were shown to react with both electrophilic and nucleophilic species.	(103)

**IAA assay**	According to this assay, Iodoacetic acid (IAA) a thiol blocking agent would react with both free thiols and protein persulfides. Subsequently, DTT would cleave the alkylated persulfide. This was followed by labeling of that particular cysteine with iodoacetamide-linked biotin (IAP). However, how this method distinguishes the persulfides from intramolecular and intermolecular disulfides and S-nitrosothiols, which would also be reduced by DTT, remains poorly understood.	(130)

**Maleimide assay**	This assay was based on the fact that *N*-ethyl maleimide, a thiol-blocking agent, would block both free thiol and persulfide. Maleimide was conjugated to Cy5. DTT was used to cleave the alkylated moieties. Fluorescence signal would decrease if the sample contains persulfides. Ratiometric decrease in fluorescence can be used for quantification.	(131)

**Biotin thiol assay**	Maleimide-PEG2-Biotin was used to alkylate both thiols and persulfides followed by binding of the proteins on an avidin column. Elution was done by DTT which cleaved the disulfide bond leaving the biotin tag bound to the column. The eluate containing the persulfidated proteins was analysed by Western blotting and LC-MS/MS.	(132)

**Pro-Per-DP –Protein persulfide detection protocol**	Iodoacteyl PEG2 Biotin was used to alkylate both free thiols and persulfides. These species were pulled down by streptavidin coated magnetic beads and subsequently reduced with DTT. The supernatant, containing the persulfidated proteins were analysed by MS.	(133)

**Tag switch assay**	This assay exploits the difference in chemical and physical properties of the thiol and the persulfide group. A thiol-blocking reagent methylsulfonyl benzothiazole, reacts with free thiol and persulfide. When compared to disulfides in proteins, the disulfide bond in persulfide adducts react strongly to nucleophiles. Subsequently, a new tag-switch reagent, biotinylated cyanoacetic acid was used to label persulfidated proteins specifically. This method has been further improved by the use of cyanoacetic acid derivatives with BODIPY moiety (CN-BOT) for labeling cells and Cy3-dye (CN-Cy3) for labeling cell lysates, respectively.	(102,134)

## Abbreviations

CBS: Cystathionine beta synthase
CSE: Cystathionine gamma lyase
3-MST: 3-mercaptopyruvate sulfurtransferase
SQR: Sulfide quinone oxidoreductase
LTP: Long term potentiation
NMDA: N-methyl-D-aspartate
GSH: Glutathione
RSV: Respiratory syncytial virus
HIV-1: Human immunodeficiency virus type 1
PLP: Pyridoxal-5-phosphate
SAM: S-adenosyl methionine
DAO: D-amino oxidase
3-MP: 3-mercaptopyruvate
ox-PTM: Oxidative posttranslational modification
ROS: Reactive oxygen species
RNS: Reactive nitrogen species
ROI: Reactive oxygen intermediates
CcO: Cytochrome c oxidase
SOD: Superoxide dismutase
OCR: Oxygen consumption rate
LC-MS: Liquid chromatography - Mass spectrometry
GAPDH: Glyceraldehyde-3-phosphate dehydrogenase
Nrf2: Nuclear factor-erythroid-2 p45 related factor 2
ARE: Antioxidant response element
Keap1: Kelch-like ECH-associated protein 1
NF-κB: Nuclear factor kappa-light-chain-enhancer of activated B cell
GC: Gas chromatography
HPLC: High pressure liquid chromatography
FAM: Fluorescein
GFP: Green fluorescent protein
pAzF: para-Azido phenylalanine
MMTS: S-methyl methanethiosulfonate
IAA: Iodoacetic acid
IAM: Iodoacetamide
DTT: Dithiothreitol
CydB: Cytochrome bd oxidase
CyoA: Cytochrome bo oxidase
MSH: Mycothiol
MSSM: Oxidized mycothiol
RNA: Ribonucleic acid
DNA: Deoxyribonucleic acid
Trx: Thioredoxin
PBMC: Peripheral blood mononuclear cells
AP-1: Activator protein 1
Sp1: Specificity protein 1
LTR: Long terminal repeat
roGFP: Redox-sensitive green fluorescent protein
NAC: N-acetylcysteine
SRB: Sulfur reducing bacteria
UC: Ulcerative colitis
TFF3: Trefoil factor 3
IRF3: Interferon regulatory factor
MAPK: Mitogen-activated protein kinase
ERK: Extracellular signal regulated kinase
JNK: c-Jun N-terminal kinase
